# Vitamin D supplementation for tuberculosis prevention: A meta-analysis

**DOI:** 10.17305/bb.2025.12527

**Published:** 2025-07-02

**Authors:** Sheng Liu, Tianyu Lin, Yanyu Pan

**Affiliations:** 1 The Second Department of Infection, 900th Hospital of PLA Joint Logistic Support Force, Fuzhou, Fujian Province, China

**Keywords:** Tuberculosis, prevention, vitamin D, supplementation, meta-analysis

## Abstract

Vitamin D plays an important role in immune regulation, prompting interest in its potential for preventing tuberculosis. However, clinical findings regarding its protective effects against tuberculosis infection and disease remain inconsistent. We conducted a systematic review and meta-analysis of randomized controlled trials (RCTs) to assess the impact of vitamin D supplementation on the prevention of tuberculosis infection and the progression to active tuberculosis. We searched PubMed, Embase, Cochrane Library, and Web of Science databases through January 2025. Eligible studies involved participants without active tuberculosis at baseline and reported outcomes related to tuberculosis. Pooled odds ratios (ORs) and 95% confidence intervals (CIs) were calculated using a random-effects model. Subgroup and sensitivity analyses were conducted, and the certainty of evidence was evaluated using the GRADE approach. Six RCTs, involving 15,677 participants, met our inclusion criteria. Compared to placebo, vitamin D supplementation did not significantly reduce the risk of tuberculosis infection (5 RCTs; OR: 0.95; 95% CI: 0.79–1.14; *P* ═ 0.55) or the development of active tuberculosis (4 RCTs; OR: 0.77; 95% CI: 0.56–1.05; *P* ═ 0.10). The certainty of evidence was moderate for both outcomes. Subgroup analyses based on baseline vitamin D levels and duration of follow-up yielded consistent results. The incidence of serious adverse events was comparable between the vitamin D and placebo groups (OR: 1.02; 95% CI: 0.76–1.38; *P* ═ 0.87), and none of the serious events were attributed to vitamin D supplementation. In conclusion, vitamin D supplementation does not significantly reduce the risk of tuberculosis infection or progression to active tuberculosis, although it is safe and well tolerated.

## Introduction

Tuberculosis remains one of the leading infectious causes of morbidity and mortality worldwide [1, 2]. According to the World Health Organization, an estimated 10.6 million people developed tuberculosis in 2021, and 1.6 million died from the disease, making it the second leading infectious killer after COVID-19 [3]. The global burden of tuberculosis is disproportionately concentrated in low- and middle-income countries, with South-East Asia and Africa experiencing the highest prevalence [4]. Beyond its immediate health impact, tuberculosis has long-term consequences, including chronic lung damage, socioeconomic hardship, and increased vulnerability to reinfection and other comorbidities [5, 6]. Children, individuals with compromised immune systems (such as those living with HIV), the elderly, and people living in crowded or under-resourced environments are especially vulnerable to infection and progression to active disease [7, 8]. Given the persistent global burden, the heightened risk among certain populations, and the limited effectiveness of current control measures in many settings, there is an urgent need to identify additional preventive strategies. Vitamin D, a fat-soluble secosteroid hormone, is essential for calcium and phosphate metabolism and bone health, but it also plays an increasingly recognized role in modulating the immune response [9, 10]. It is synthesized in the skin upon exposure to ultraviolet B radiation or obtained through diet and supplements [11]. Once activated to its hormonal form, 1,25-dihydroxyvitamin D, it binds to the vitamin D receptor (VDR), which is expressed in various cell types, including immune cells such as monocytes, macrophages, and dendritic cells [12]. In the context of tuberculosis, vitamin D enhances the antimicrobial activity of macrophages, promotes the production of cathelicidin and other antimicrobial peptides, and supports autophagy and phagolysosome fusion—mechanisms critical for host defense against Mycobacterium tuberculosis [13, 14]. It also regulates the adaptive immune system by modulating T-cell differentiation and cytokine responses, helping to maintain immune balance [15]. Observational studies have consistently shown an association between low serum levels of 25-hydroxyvitamin D and increased susceptibility to tuberculosis infection and progression. Individuals with active or latent tuberculosis tend to have lower circulating vitamin D levels compared to healthy controls [16–19]. Moreover, people with vitamin D deficiency may be more likely to progress from latent infection to active disease, especially in the presence of other risk factors such as HIV infection or malnutrition [18, 20, 21]. These findings have fueled growing interest in whether vitamin D supplementation could serve as a cost-effective and safe strategy to reduce tuberculosis risk [22, 23]. Despite its biological plausibility and supportive observational data, randomized controlled trials (RCTs) examining the efficacy of vitamin D supplementation in preventing tuberculosis infection or disease have yielded inconsistent results [24–29]. Differences in study populations, baseline vitamin D status, supplementation regimens, and the outcomes measured have likely contributed to the variability [24–29]. As a result, there is currently no consensus on whether routine vitamin D supplementation should be recommended as part of tuberculosis prevention strategies, particularly for high-risk populations such as children, individuals with HIV, or those living in endemic areas [30]. Given these uncertainties, we conducted a meta-analysis to systematically evaluate the impact of vitamin D supplementation on the risk of tuberculosis infection and the development of active disease.

## Materials and methods

During the design and implementation of this study, we followed the guidelines outlined by Preferred Reporting Items for Systematic Reviews and Meta-Analyses (PRISMA) [31, 32] and the Cochrane Handbook [33]. The meta-analysis protocol was registered with PROSPERO under the identifier CRD420251004949.

### Study inclusion and exclusion criteria

This meta-analysis included studies that met the inclusion criteria specified in the PICOS principle.

P (Patients): Children or adults without active tuberculosis at baseline.

I (Intervention): Vitamin D supplementation administered in various dosages and durations.

C (Control): Standard treatment, no treatment, or controls with similar appearance and administration route to the intervention.

O (Outcome): Incident tuberculosis infection or development of active tuberculosis, and the methods for the diagnosis of tuberculosis infection or active tuberculosis were consistent with the criteria used in the original studies.

S (Study design): RCTs.

Excluded from the analysis were reviews, editorials, preclinical studies, studies not designed as RCTs, studies involving patients with active tuberculosis, those not including vitamin D supplementation as an intervention, and those not reporting the outcomes of interest. If multiple studies with overlapping patient populations were identified, the study with the largest sample size was included in the meta-analysis.

### Database search

The Medline (PubMed), Embase (Ovid), CENTRAL (Cochrane Library), and Web of Science databases were searched using the following combination of terms: (1) “vitamin D” OR “vitamin D2” OR “vitamin D3” OR “cholecalciferol” OR “ergocalciferol” OR “alphacalcidol” OR “alfacalcidol” OR “calcitriol” OR “paricalcitol” OR “doxerocalciferol”; and (2) “tuberculosis” OR “Mycobacterium tuberculosis” OR “tuberculous.” The search was limited to clinical studies in humans. Only studies involving human subjects and published in English were included. The complete search strategy for each database is provided in Supplemental File 1. Additionally, references from related reviews and original articles were screened during the final database search. The final search was conducted on January 29, 2025.

### Data collection and quality evaluation

Two authors independently conducted database searches, data collection, and quality assessments. In cases of disagreement, discussions were held with the corresponding author to reach consensus. The collected data covered various aspects, including general study information (e.g., first author, publication year, and study country), study design (double-blind or single-blind), participant characteristics (general health status, number of participants, mean age, sex, and baseline serum levels of 25-hydroxyvitamin D [25(OH)D]), details of the vitamin D supplementation intervention (administration method—oral or transdermal—dosage, and treatment frequency), control group details, follow-up duration, and definitions and outcomes related to tuberculosis infection. The quality of the included RCTs was assessed using the Cochrane Risk of Bias Tool [33], which evaluates factors such as random sequence generation, allocation concealment, blinding of participants and outcome assessors, handling of incomplete outcome data, selective reporting, and other potential sources of bias. Additionally, two reviewers assessed the certainty of the evidence using the GRADE (Grading of Recommendations, Assessment, Development and Evaluation) system, which considers risk of bias, inconsistency, indirectness, imprecision, and publication bias [34]. The certainty of evidence was categorized as very low, low, moderate, or high. Any disagreements were resolved through discussion with the corresponding author.

### Statistical analysis

The influence of vitamin D supplementation on the risk of tuberculosis infection and the development of active tuberculosis, compared to controls, was summarized using odds ratios (ORs) and corresponding 95% confidence intervals (CIs) [33]. We also compared the incidence of serious adverse events (SAEs) between the two groups, as defined by the criteria used in the original studies. These typically included fatal or non-fatal events leading to discontinuation of the study medication, as well as other monitored safety concerns such as hypercalcemia, hypervitaminosis D, and renal stones. Heterogeneity was assessed using the Cochrane *Q* test [33], and the *I^2^* statistic was calculated, with values of < 25%, 25%–75%, and > 75% indicating low, moderate, and high heterogeneity, respectively [35]. A random-effects model was used to pool results, as it accounts for potential heterogeneity across studies [33]. Sensitivity analysis was conducted by excluding one dataset at a time to evaluate the robustness of the findings [33]. Predefined subgroup analyses were also performed to examine the influence of study characteristics on the outcomes, such as baseline serum 25(OH)D levels and follow-up durations. Medians of continuous variables were used as cutoff values to define subgroups. Publication bias was assessed through visual inspection of funnel plots and Egger’s regression asymmetry test [36]. A *P* < 0.05 was considered statistically significant. Statistical analyses were conducted using RevMan (version 5.1; Cochrane, Oxford, UK) and Stata (version 17.0; StataCorp, College Station, TX, USA).

**Table 1:** Characteristics of the included RCTs

**Study**	**Country**	**Design**	**Participant characteristics**	**No. of participants**	**Mean age (years)**	**Male (%)**	**Baseline serum 25(OH)D**	**Intervention**	**Control**	**Follow-up duration (months)**	**Diagnosis of TB**
Ganmaa et al., 2012	Mongolia	R, DB, PC	Schoolchildren aged 12–15 years in Ulaanbaatar, TST-negative at baseline	117	13.1	49.2	Mean: 7 ng/mL (all < 20 ng/mL; 82% < 10 ng/mL)	800 IU/day vitamin D_3_ for 6 months	Placebo capsule, same appearance	6	TB infection as indicated by TST conversion (≥10 mm) and confirmed with T-SPOT. TB if converted
Yani et al., 2018	Indonesia	R, DB, PC	Healthy children (under 5 years) with recent TB contact and TST-negative at baseline	66	NR (<5)	NR	<30 ng/mL	Two high single doses of vitamin D_3_, 6 weeks apart	Placebo	3	TB infection as indicated by TST conversion (induration >10 mm at 12 weeks)
Sudfeld et al., 2020	Tanzania	R, DB, PC	Adults (≥18 years) with HIV initiating ART and serum 25(OH)D < 30 ng/mL	3639	38.7	32	<30 ng/mL in all participants (48% insufficient, 46% moderately deficient, 6–8% severely deficient)	50,000 IU Vit D_3_ weekly for 4 weeks, then 2,000 IU daily for 12 months	Identical placebo	12	Active TB as indicated by clinical symptoms + sputum AFB smear and/or chest X-ray; GeneXpert used later in the study
Ganmaa et al., 2020	Mongolia	R, DB, PC	Schoolchildren aged 6–13 years, QFT-negative at baseline, 95.6% had vitamin D <20 ng/mL	8819	9.4	50.7	Mean: 11.9 ng/mL; 95.6% <20 ng/mL; 31.8% <10 ng/mL	Weekly oral 14,000 IU vitamin D_3_ for 36 months	Placebo	36	TB infection as indicated by QFT-Gold conversion; clinical diagnosis for active TB
Dude et al., 2022	India	R, DB, PC	Schoolchildren (aged 6–11 years), with negative QFT-Plus at baseline, no histories of TB	1354	8.9	47.6	Mean: 28.5 ng/mL	Weekly vitamin D_3_ 350 µg (14,000 IU) for 36 months	Placebo	36	TB infection as indicated by QFT-Plus test; clinical diagnosis for active TB
Middelkoop et al., 2023	South Africa	R, DB, PC	Healthy children aged 6–11 years with negative QFT-Plus at baseline, no chronic illness	1682	8.9	47.6	Mean 71.2 nmol/L (28.5 ng/mL); 63.2% <75 nmol/L	Weekly 10,000 IU vitamin D_3_ for 3 years	Placebo, identical soft-gel capsule	36	TB infection as indicated by QFT-Plus assay conversion; active TB assessed by clinical evaluation

Abbreviations: 25(OH)D: 25-hydroxyvitamin D; AFB: Acid-fast bacilli; ART: Antiretroviral therapy; DB: Double-blind; IU: International units; NR: Not reported; PC: Placebo-controlled; QFT: QuantiFERON-TB Gold; QFT-Plus: QuantiFERON-TB Gold Plus; R: randomized; TB: Tuberculosis; TST: Tuberculin skin test.

## Results

### Literature search

**Figure 1.**
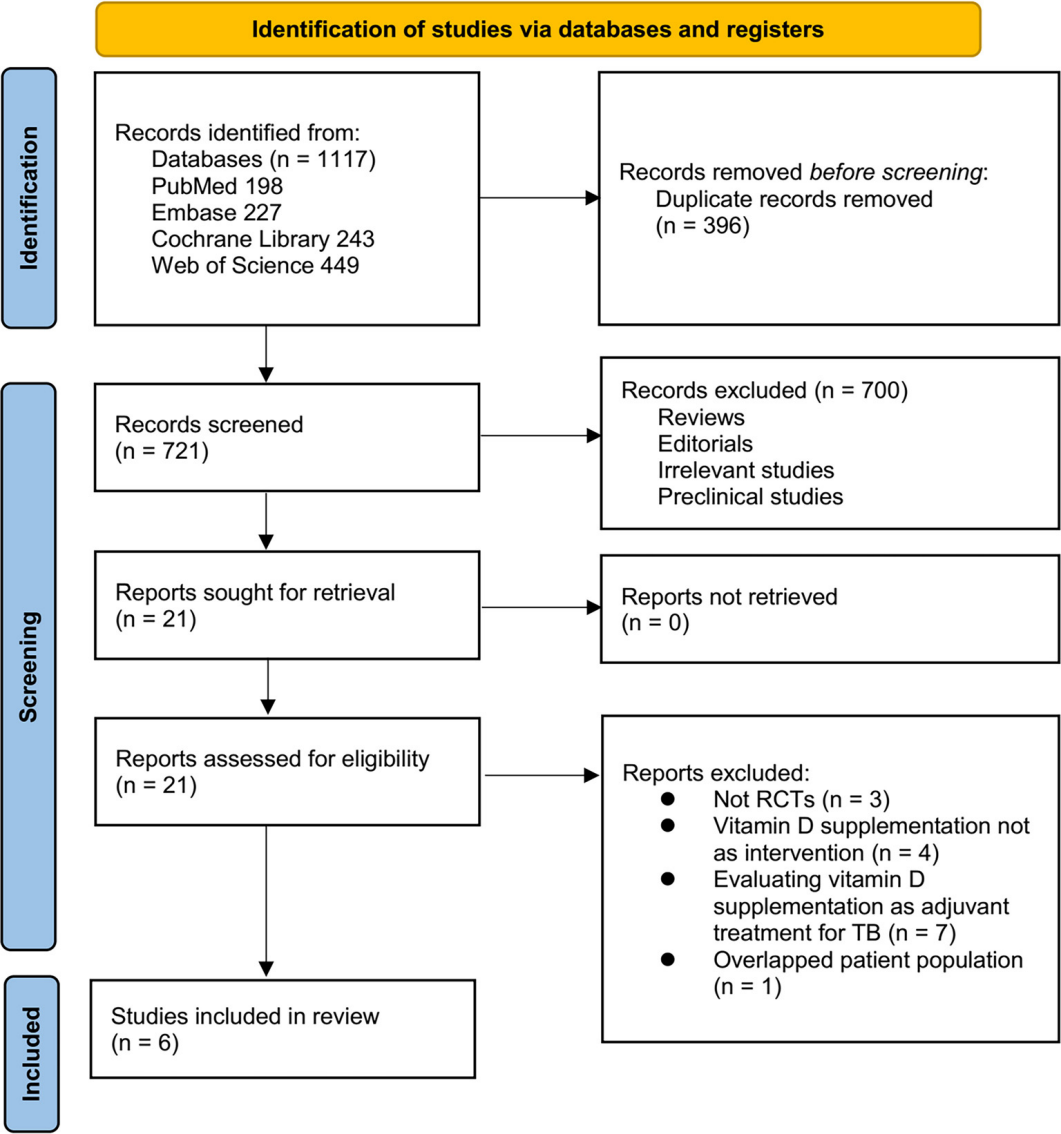
Flowchart for the literature search and study inclusion.

Figure 1 presents a flowchart outlining the process of database searching and study selection for inclusion. Initially, 1117 articles were identified through the database search. After removing 396 duplicate records, 721 articles remained. Of these, 700 were excluded following a review of titles and abstracts, primarily due to a lack of relevance to the objective of the present meta-analysis. A full-text assessment of the remaining 21 articles led to the exclusion of 15 studies for reasons detailed in Figure 1. Ultimately, six RCTs [24–29] were deemed suitable for quantitative analysis.

### Study characteristics and data quality

An overview of the included studies is provided in Table 1. These six randomized, double-blind, placebo-controlled trials were conducted in Mongolia [24, 26], Indonesia [25], Tanzania [27], India [28], and South Africa [29], and were published between 2012 and 2023. The studies enrolled both children (ages ranging from under 5 to 15 years) and adults (≥18 years) without active tuberculosis at baseline. A total of 15,677 participants were included, with mean ages ranging from under 5–38.7 years, and the proportion of male participants ranging from 32.0% to 50.7%. Notably, although the title of the Dude et al. (2022) [28] study refers to “TB recurrence,” the trial exclusively enrolled TB-naïve children with no history of infection, aligning with the preventive focus of the present meta-analysis. In [25], while the exact mean age was not reported, all participants were confirmed to be under five years old. Given their young age and the potential influence of BCG vaccination, TST-based diagnoses in this subgroup may have reduced specificity. Four studies included participants with baseline serum 25(OH)D levels < 30 ng/mL [24–27], while two studies enrolled participants with serum levels either < 30 ng/mL or ≥ 30 ng/mL [28, 29]. Vitamin D_3_ supplementation was administered orally in varying doses and regimens—including daily, weekly, or high single doses—over durations ranging from 3–36 months. Placebo controls were matched to the interventions in appearance and administration. Tuberculosis infection was diagnosed using the tuberculin skin test (TST) in two studies [24, 25] and QuantiFERON-TB Gold (QFT) or QFT-Plus in three studies [26, 28, 29]. Active tuberculosis was diagnosed in four studies [26–29] using clinical symptoms, radiological findings, or microbiological tests. Study quality assessments are presented in Table 2. All included studies were judged to have a low risk of bias across all domains, with the exception of two studies [25, 28], which had unclear risk in the domains of random sequence generation and allocation concealment due to insufficient reporting.

**Table 2:** Study quality evaluation via the Cochrane risk of bias tool

**Study**	**Random sequence generation**	**Allocation concealment**	**Blinding of participants**	**Blinding of outcome assessment**	**Incomplete outcome data addressed**	**Selective reporting**	**Other sources of bias**
Ganmaa et al., 2012	Low risk	Low risk	Low risk	Low risk	Low risk	Low risk	Low risk
Yani et al., 2018	Unclear	Unclear	Low risk	Low risk	Low risk	Low risk	Low risk
Sudfeld et al., 2020	Low risk	Low risk	Low risk	Low risk	Low risk	Low risk	Low risk
Ganmaa et al., 2020	Low risk	Low risk	Low risk	Low risk	Low risk	Low risk	Low risk
Dude et al., 2022	Unclear	Unclear	Low risk	Low risk	Low risk	Low risk	Low risk
Middelkoop et al., 2023	Low risk	Low risk	Low risk	Low risk	Low risk	Low risk	Low risk

**Figure 2.**
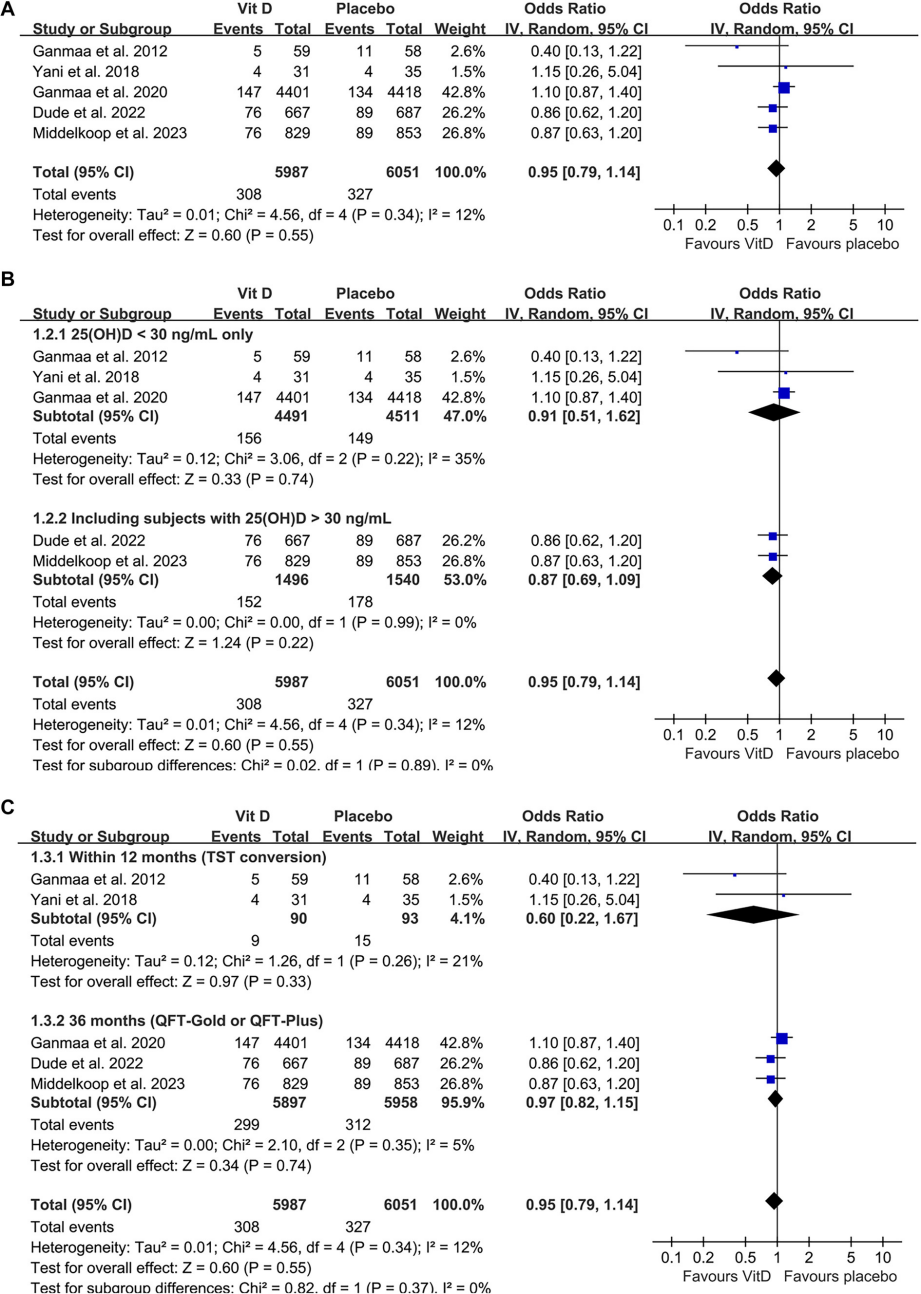
**Forest plots for the meta-analysis evaluating the influence of vitamin D supplementation on the incidence of tuberculosis infection.** (A) Overall meta-analysis; (B) Subgroup analysis according to the baseline serum 25(OH)D level; (C) Subgroup analysis according to follow-up durations. Abbreviation: CI: Confidence interval.

### Influence of vitamin D supplementation on tuberculosis infection

Five studies assessed the impact of vitamin D supplementation on the risk of tuberculosis infection [24–26, 28, 29], with mild heterogeneity observed (*P* for Cochrane *Q* test ═ 0.34; *I^2^* ═ 12%). Pooled results indicated that, overall, vitamin D supplementation did not significantly reduce the risk of tuberculosis infection compared to placebo (OR: 0.95, 95% CI: 0.79–1.14, *P* ═ 0.55; Figure 2A). The certainty of the evidence, summarized using the GRADE system, is presented in Table 3. We downgraded the evidence by one level due to potential publication bias stemming from the limited number of included studies, and judged the evidence to be of moderate certainty. Sensitivity analysis, conducted by excluding one dataset at a time, showed consistent results (OR: 0.84–0.98; all *P* values > 0.05). Subsequent subgroup analyses also yielded similar outcomes. Studies enrolling only participants with baseline 25(OH)D levels < 30 ng/mL were comparable to those including participants with levels < or ≥30 ng/mL (OR: 0.91 vs 0.87; *P* for subgroup difference ═ 0.89; Figure 2B). Additionally, similar findings were observed between studies with a follow-up of up to 12 months, where tuberculosis infection was defined by TST conversion, and those with a 36-month follow-up, where infection was defined by QFT conversion (OR: 0.60 vs 0.97; *P* for subgroup difference ═ 0.37; Figure 2C).

**Table 3:** Summarized certainty of evidence using the GRADE system

**Outcome**	**Quality assessment**							**Absolute effect** **OR (95% CI)**	**Quality**
	**No. of studies**	**Design**	**Risk of bias**	**Inconsistency**	**Indirectness**	**Imprecision**	**Other considerations**		
OR for TB infection	5	RCTs	No serious risk of bias	No serious inconsistency	No serious indirectness	No serious imprecision	Possible publication bias due to limited number of studies included	0.95 (0.79 to 1.14)	⊕⊕⊕O MODERATE
OR for active TB	4	RCTs	No serious risk of bias	No serious inconsistency	No serious indirectness	No serious imprecision	Possible publication bias due to limited number of studies included	0.77 (0.56 to 1.05)	⊕⊕⊕O MODERATE
OR for severe AEs	3	RCTs	No serious risk of bias	No serious inconsistency	No serious indirectness	No serious imprecision	Possible publication bias due to limited number of studies included	1.02 (0.76 to 1.38)	⊕⊕⊕O MODERATE

Abbreviations: AE: Adverse event; CI: Confidence interval; OR: Odds ratio; RCT: Randomized controlled trial; TB: Tuberculosis.

### Influence of vitamin D supplementation on the development active tuberculosis

**Figure 3.**
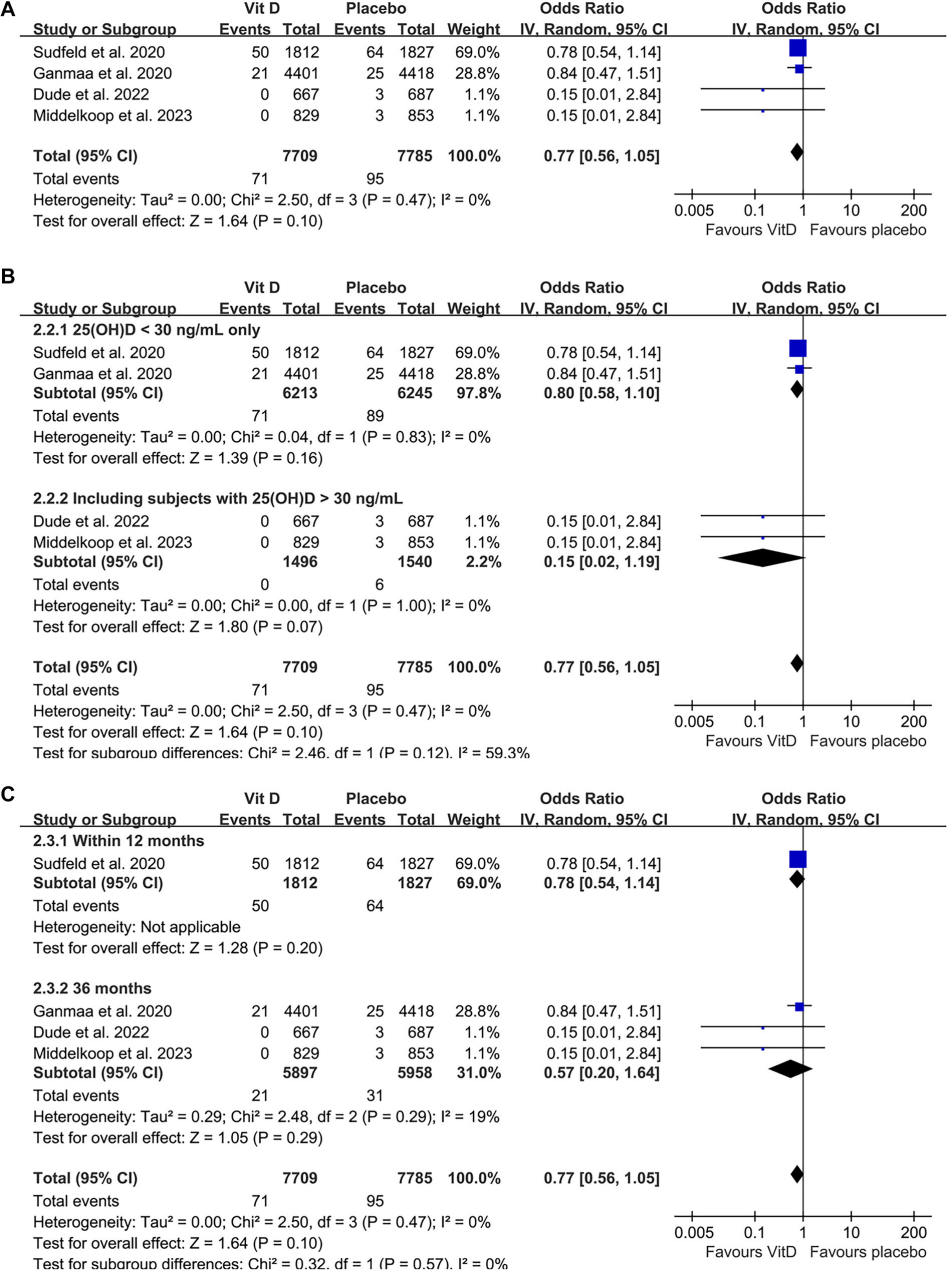
**Forest plots for the meta-analysis evaluating the influence of vitamin D supplementation on the incidence of active tuberculosis.** (A) Overall meta-analysis; (B) Subgroup analysis according to the baseline serum 25(OH)D level; (C) Subgroup analysis according to follow-up durations. Abbreviation: CI: Confidence interval.

The results of a meta-analysis involving four studies [26–29] suggested that vitamin D supplementation did not significantly reduce the incidence of active tuberculosis compared to placebo (OR: 0.77; 95% CI: 0.56–1.05; *P* ═ 0.10; Figure 3A), with no significant heterogeneity observed (Cochrane *Q* test *P* ═ 0.47; *I^2^* ═ 0%). The certainty of the evidence, summarized in Table 3, was rated as moderate due to potential publication bias stemming from the limited number of included studies. Sensitivity analysis, conducted by omitting one dataset at a time, did not meaningfully alter the results (OR range: 0.57–0.78; all > 0.05). Similar findings were observed in subgroup analyses: studies that included only participants with baseline 25(OH)D < 30 ng/mL showed results consistent with those including participants with baseline levels both < and ≥ 30 ng/mL (OR: 0.80 vs 0.15; *P* for subgroup difference ═ 0.12; Figure 3B). Likewise, no significant differences were observed between studies with follow-up durations of 12 and 36 months (OR: 0.78 vs 0.57; *P* for subgroup difference ═ 0.57; Figure 3C).

### Incidence of adverse events

**Figure 4.**
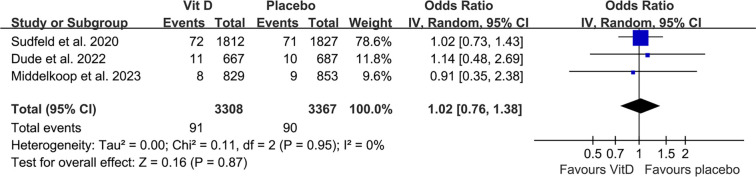
**Forest plots for the meta-analysis evaluating the incidence of severe adverse events (AEs)**. Abbreviation: CI: Confidence interval.

Across the included studies, SAEs were rare and occurred at similar rates in both the vitamin D and placebo groups [27–29]. When reported, SAEs were primarily non-fatal hospitalizations or isolated deaths, with none attributed to vitamin D supplementation. These findings suggest that vitamin D is generally safe and well tolerated for tuberculosis prevention. Pooled results from three studies [27–29] showed comparable SAE incidence between participants receiving vitamin D supplementation and those receiving a placebo (OR: 1.02, 95% CI: 0.76–1.38, *P* ═ 0.87; Figure 4), with no significant heterogeneity (Cochrane Q test *P* ═ 0.95; *I^2^* ═ 0%). The certainty of evidence, summarized in Table 3, was rated as moderate due to the potential for publication bias arising from the limited number of included studies.

### Publication bias

**Figure 5.**
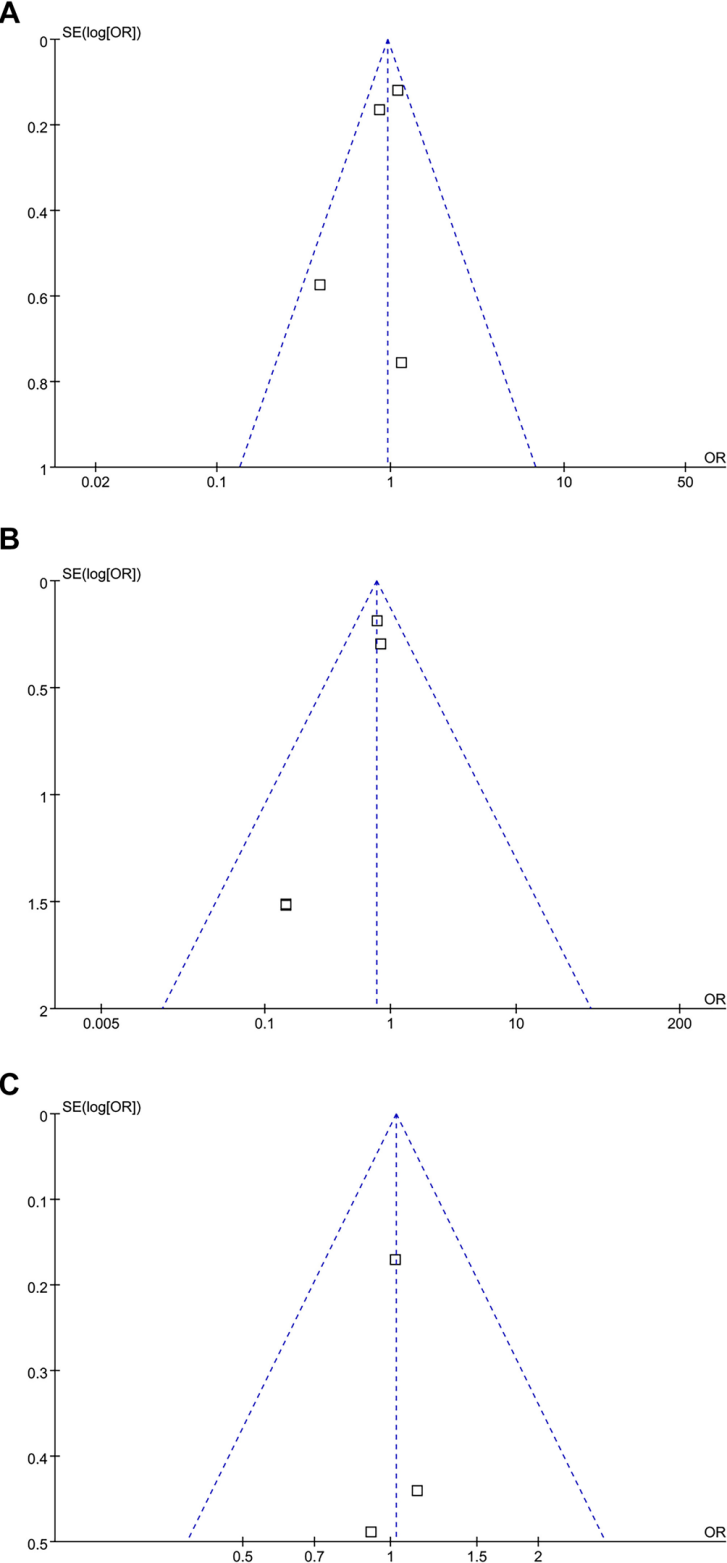
**Funnel plots evaluating the publication bias underlying the meta-analyses.** (A) Funnel plots for the meta-analysis of the incidence of tuberculosis infection; (B) Funnel plots for the meta-analysis of the incidence of active tuberculosis; (C) Funnel plots for the meta-analysis of the incidence severe AEs. Abbreviation: AEs: Adverse events.

The funnel plots for the meta-analyses comparing the effects of vitamin D supplementation on tuberculosis infection, progression to active tuberculosis, and SAEs vs placebo are shown in Figure 5A–5C. These plots appear symmetrical upon visual inspection, suggesting a low risk of publication bias. Egger’s regression test could not be performed due to the limited number of included studies (three to five) for these outcomes.

## Discussion

This meta-analysis of six high-quality RCTs, involving over 15,000 participants from diverse geographic and demographic backgrounds, found that vitamin D supplementation did not significantly reduce the risk of TB infection or progression to active TB compared to placebo. Pooled results showed no statistically significant effect on either outcome, with consistent findings across sensitivity and subgroup analyses. Vitamin D supplementation was also found to be safe and well tolerated, with comparable rates of SAEs between intervention and control groups and no events attributable to the supplementation. These findings suggest that, despite strong biological plausibility and supportive evidence from observational studies, vitamin D supplementation alone may be insufficient to prevent TB infection or the development of active disease. Several physiological and immunological factors may explain this disconnect. Although vitamin D exerts known immunomodulatory effects—such as enhancing macrophage activation, upregulating antimicrobial peptides like cathelicidin, and supporting autophagy and phagolysosome fusion—these innate immune responses may not be robust enough to prevent infection or eliminate Mycobacterium tuberculosis following exposure [37, 38]. Moreover, TB is a complex disease shaped by numerous host, pathogen, and environmental variables [39]. In settings of high pathogen load or immunosuppressive conditions, any protective effects of vitamin D may be overwhelmed [40, 41]. It is therefore possible that vitamin D plays more of an adjunctive role in host defense—supporting immune function but not providing sufficient protection on its own, particularly in individuals without profound deficiency or in the absence of other complementary interventions [42]. Subgroup analyses based on baseline vitamin D status and follow-up duration offered further insights. Results were similar between studies enrolling participants with serum 25(OH)D levels consistently below 30 ng/mL and those with a broader range of baseline levels, suggesting that supplementation does not confer additional protection even in deficient populations. Similarly, the null effect remained consistent across studies using different diagnostic definitions of TB infection—namely, TST conversion over shorter follow-up periods and interferon-gamma release assay (IGRA), such as QuantiFERON-TB (QFT), conversion over longer durations. Notably, TST may overestimate infection rates in BCG-vaccinated populations due to cross-reactivity, while IGRAs offer greater specificity [43, 44]. Interestingly, although not statistically significant (*P* for subgroup difference ═ 0.37), the subgroup analysis showed a numerically lower OR in studies using TST (OR: 0.60) compared to those using QFT/IGRA (OR: 0.97). This trend may reflect differences in assay sensitivity or specificity—particularly in BCG-vaccinated individuals—and warrants further investigation in future studies employing harmonized diagnostic protocols. Overall, these subgroup findings reinforce the robustness of the null effect and suggest that the lack of benefit is not attributable to specific study designs, populations, or diagnostic methods. This meta-analysis has several notable strengths. First, we conducted a comprehensive and up-to-date literature search across multiple databases, applying strict inclusion criteria limited to double-blind, placebo-controlled RCTs—the gold standard for evaluating intervention efficacy. Second, the included studies encompassed diverse populations, from young children to adults with HIV, enhancing the generalizability of our findings. Third, the consistent results across sensitivity and subgroup analyses lend confidence to the stability of the findings. Fourth, the risk of bias was judged to be low across most domains in all included studies, further supporting the internal validity of the meta-analysis. However, several limitations should also be acknowledged. The number of available studies per outcome was relatively small, which may limit statistical power and precision. While we rated the certainty of evidence as moderate for all outcomes, this reflects a balance between the low risk of bias and consistent findings, and limitations due to the small number of studies, potential undetected publication bias, and imprecise effect estimates. The limited number of studies also precluded meaningful meta-regression or more detailed subgroup analyses. For example, although investigating varying degrees of baseline vitamin D deficiency could provide additional insights, this was not feasible due to inconsistent reporting and the absence of stratified outcome data. Most studies reported only mean or median baseline 25(OH)D levels, with few providing subgroup results based on established deficiency thresholds, such as < 30 ng/mL (insufficient) or < 20 ng/mL (deficient). This represents a gap in the literature and underscores the need for future trials to incorporate and report more detailed stratifications of vitamin D status. Substantial heterogeneity in participant characteristics (e.g., age and comorbidities), baseline vitamin D levels, dosing regimens (including dose, frequency, and duration), and definitions of tuberculosis outcomes may have masked subgroup-specific effects. Other potentially important contributors to heterogeneity—such as host genetic differences (e.g., VDR polymorphisms), local TB transmission dynamics, and variations in nutritional or immune status—could not be assessed due to the limited number of studies and lack of stratified or individual participant-level data. Although a dose–response relationship is of clinical interest, such analysis was not feasible due to the absence of dose-stratified outcome data, considerable variability in dosing regimens (daily, weekly, or bolus), inconsistent reporting of participant body weight, and too few studies per outcome to permit reliable meta-regression. As shown in Supplementary File 2, which details individual dosing regimens and associated effect estimates, no consistent pattern of benefit was observed across different vitamin D schedules. However, this clinical heterogeneity may have diluted potential protective effects in more responsive subgroups. Wide variation in baseline 25(OH)D levels and differing definitions of TB outcomes further complicate interpretation and may have contributed to the overall null effect. These findings highlight the need for future trials in well-characterized populations, with more detailed reporting to better elucidate population-specific responses to vitamin D supplementation. Another important limitation is the restricted geographic representation of the included studies, which were primarily conducted in Asia and Africa. Data from Latin America, Eastern Europe, and other high-burden regions are lacking. Variations in sunlight exposure, dietary patterns, nutritional status, TB prevalence, and healthcare infrastructure across settings may influence both baseline vitamin D status and the efficacy of supplementation. Future research should aim to include more geographically diverse populations to improve generalizability. Lastly, the possibility of publication bias cannot be excluded, given the small number of available trials and the lack of unpublished or negative studies [45]. While visual inspection of funnel plots suggested low publication bias, the reliability of this assessment is limited due to the small number of studies. Formal tests such as Egger’s regression are underpowered when fewer than 10 studies are available per outcome, increasing the risk of undetected bias. Therefore, the potential for publication bias—particularly from small, negative, or unpublished trials—remains an important limitation of this meta-analysis. From a clinical perspective, these findings do not support the routine use of vitamin D supplementation solely for the prevention of tuberculosis in the general population or in high-risk groups such as children with tuberculosis contact or people living with HIV. However, personalized approaches that consider individual risk profiles—such as profound vitamin D deficiency, immunosuppression, or high endemic exposure—may still hold value. Assessing baseline 25(OH)D levels and selectively supplementing individuals at greatest risk may offer a more effective and pragmatic strategy in clinical practice. In addition, vitamin D supplementation remains important for musculoskeletal health and correction of deficiency [46], but its role in tuberculosis prevention appears limited based on current evidence [47]. These results also reinforce the complexity of tuberculosis prevention, which likely requires a multifaceted approach including vaccination, chemoprophylaxis in high-risk groups, improved living conditions, and control of comorbid conditions such as HIV [48]. Future research should aim to address the remaining uncertainties. Large-scale trials focused on specific subpopulations—such as individuals with profound vitamin D deficiency, genetic variants affecting vitamin D metabolism or receptor function, or those with significant immunosuppression—may help identify groups who might benefit more from supplementation [49]. Trials should also explore optimized dosing strategies, including higher or more prolonged regimens, as well as the potential synergistic effects of combining vitamin D with other preventive interventions [49]. In our meta-analysis, dosing schedules varied widely across studies, including daily, weekly, and high single-dose bolus regimens. However, no clear trend toward greater efficacy was observed for any particular regimen. Given this variability and the absence of stratified efficacy results by dosing strategy in the included trials, the comparative effectiveness of different vitamin D supplementation approaches remains an open question for future research. Additionally, mechanistic studies exploring the interaction between vitamin D signaling and host-pathogen dynamics in tuberculosis are warranted to better understand the biological boundaries of its protective effects. Given the limited number of high-quality RCTs currently available, there is a clear need for larger, well-designed, multicenter trials employing standardized dosing regimens, diagnostic criteria, and follow-up durations. Such studies would enhance statistical power, minimize heterogeneity, and provide more definitive conclusions regarding the potential preventive effects of vitamin D against tuberculosis. Additionally, future trials may benefit from focusing on subpopulations with profound vitamin D deficiency or specific genetic variants related to the VDR, which may modulate the immune response to Mycobacterium tuberculosis. Such stratified approaches could improve trial efficiency and yield more clinically actionable insights. Future trials should also be adequately powered to detect a small-to-moderate protective effect (e.g., OR ≤ 0.80), with consideration of sample size calculations to ensure sufficient precision. For tuberculosis infection (annual risk ∼4.0%), a sample size of approximately 9,200 participants per group would be needed to detect an OR of 0.80 with 80% power and α ═ 0.05. For active tuberculosis (annual risk ∼0.5%), more than 70,000 participants per group would be required. These figures emphasize the need for large, multicenter trials to reliably detect modest preventive effects. Finally, to enhance comparability and support future meta-analyses, the development and adoption of a core outcome set—including standardized clinical endpoints for tuberculosis infection and progression, as well as harmonized immunological biomarkers—is strongly encouraged.

## Conclusion

In conclusion, this comprehensive meta-analysis found that vitamin D supplementation does not significantly reduce the overall incidence of tuberculosis infection or progression to active disease, although it is safe and well tolerated. However, certain high-risk groups—such as individuals with severe vitamin D deficiency, immunosuppression (e.g., HIV), or specific genetic profiles—may still benefit. These findings underscore the importance of comprehensive preventive strategies for tuberculosis control and highlight the need for further targeted research in vulnerable populations.

## Supplemental data

**Supplemental file 1.** Detailed search syntax for each database

**PubMed**

((“Vitamin D”[Mesh] OR “Cholecalciferol”[Mesh] OR “Ergocalciferols”[Mesh] OR “Calcitriol”[Mesh] OR “Alfacalcidol”[Supplementary Concept] OR “Paricalcitol”[Supplementary Concept] OR “Vitamin D” OR “Vitamin D2” OR “Vitamin D3” OR “Cholecalciferol” OR “Ergocalciferol” OR “Alphacalcidol” OR “Alfacalcidol” OR “Calcitriol” OR “Paricalcitol” OR “Doxercalciferol”)) AND ((“Tuberculosis”[Mesh] OR “Mycobacterium tuberculosis”[Mesh] OR “Tuberculosis, Pulmonary”[Mesh] OR “Tuberculous” OR “Tuberculosis” OR “Mycobacterium tuberculosis”))

**Embase**

(‘vitamin D’/exp OR ‘cholecalciferol’/exp OR ‘ergocalciferol’/exp OR ‘calcitriol’/exp OR ‘alfacalcidol’/exp OR ‘paricalcitol’/exp OR ‘vitamin D’ OR ‘vitamin D2’ OR ‘vitamin D3’ OR ‘cholecalciferol’ OR ‘ergocalciferol’ OR ‘alfacalcidol’ OR ‘alphacalcidol’ OR ‘calcitriol’ OR ‘paricalcitol’ OR ‘doxercalciferol’) AND (‘tuberculosis’/exp OR ‘mycobacterium tuberculosis’/exp OR ‘tuberculous’ OR ‘tuberculosis’ OR ‘mycobacterium tuberculosis’)

**Cochrane Library**

(“Vitamin D” OR “Vitamin D2” OR “Vitamin D3” OR “Cholecalciferol” OR “Ergocalciferol” OR “Alphacalcidol” OR “Alfacalcidol” OR “Calcitriol” OR “Paricalcitol” OR “Doxercalciferol”) AND (“Tuberculosis” OR “Mycobacterium tuberculosis” OR “Tuberculous”)

**Web of Science**

TS=(“Vitamin D” OR “Vitamin D2” OR “Vitamin D3” OR “Cholecalciferol” OR “Ergocalciferol” OR “Alphacalcidol” OR “Alfacalcidol” OR “Calcitriol” OR “Paricalcitol” OR “Doxercalciferol”) AND TS=(“Tuberculosis” OR “Mycobacterium tuberculosis” OR “Tuberculous”)

**Supplemental file 2:** Summary of dosing regimens and study-level effect estimates

**Study**	**Dosing regimen**	**Outcome**	**OR (95% CI)**
Ganmaa et al., 2012	800 IU/day for 6 months	TB infection	0.91 (0.37–2.24)
Yani et al., 2018	Two high single doses, 6 weeks apart	TB infection	0.60 (0.18–1.99)
Ganmaa et al., 2020	14,000 IU/week for 36 months	TB infection	0.95 (0.76–1.19)
Ganmaa et al., 2020	14,000 IU/week for 36 months	Active TB	0.89 (0.40–1.97)
Sudfeld et al., 2020	50,000 IU/week × 4 wks → 2000 IU/day (12 mo)	Active TB	0.80 (0.46–1.39)
Dude et al., 2022	14,000 IU/week for 36 months	TB infection	0.87 (0.41–1.85)
Dude et al., 2022	14,000 IU/week for 36 months	Active TB	0.65 (0.17–2.43)
Middelkoop et al., 2023	10,000 IU/week for 36 months	TB infection	1.02 (0.70–1.49)
Middelkoop et al., 2023	10,000 IU/week for 36 months	Active TB	0.69 (0.30–1.59)

## Data Availability

The data that support the findings of this study are available from the corresponding author upon reasonable request.
